# Gallic Acid Loaded Alginate‐Gelatin Beads for Potential Bone Tissue Engineering Applications

**DOI:** 10.1002/bip.70033

**Published:** 2025-06-04

**Authors:** Mehmet Ali Karaca, Ali Reza Kamali, Bita Erin Kamali, Bilge Temiz, Furkan Özben, Duygu Ege, Hale Saybaşılı

**Affiliations:** ^1^ Institute of Biomedical Engineering, Boğaziçi University Istanbul Turkey; ^2^ Energy and Environmental Materials Research Centre (E^2^MC), School of Metallurgy, Northeastern University Shenyang China; ^3^ Cambridge Silicon Age Centre for Innovation Cambridge UK; ^4^ UCL Cancer Institute, University College London London UK

**Keywords:** alginate‐gelatin beads, bone tissue engineering, encapsulation, gallic acid, MC3T3‐E1

## Abstract

In this study, alginate/gelatin (AL/GEL) spherical beads are prepared and encapsulated with 1 wt % of needle‐shaped gallic acid (GA) crystals to develop a drug delivery system. The % encapsulation efficiency of GA into AL/GEL beads, its release rate, and the stability of the beads are evaluated, followed by cytocompatibility studies. The interactions between GA, AL, and GEL are examined by using FTIR. Morphological observations reveal that increasing the GEL concentration above 0.4 wt.% possibly hinders the binding of calcium ions with the carboxylate groups of AL, resulting in the formation of beads with larger diameters. In contrast, the bead diameter decreases with the incorporation of GA due to hydrogen bonding. EDX analysis of GA‐loaded AL/GEL beads indicated that GA binds to the GEL‐rich region. Furthermore, EDX analysis of mineralized beads demonstrated that GA enhanced calcium deposition near the alginate‐rich region. In vitro studies demonstrate that AL/GEL beads loaded with ≤ 0.5 (wt.) % GA are cytocompatible and MC3T3‐E1 murine pre‐osteoblast cells proliferated over a 5‐day period. Overall, the prepared beads show potential as a drug delivery system for bone regeneration applications.

## Graphical abstract

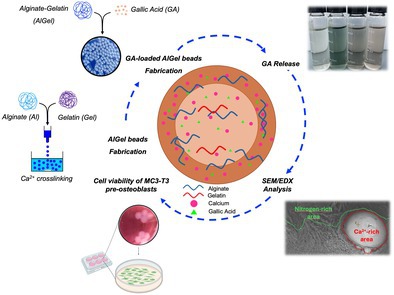

## 1 Introduction

Bone fracture resulting from bone disease significantly affects the daily life of millions of people each year, especially those over the age of 50 [1]. As people age, the number of stem cells decreases within the body, and age‐related decline in regeneration power can slow down bone healing [2]. The encapsulation of bioactive molecules and their targeted delivery of these compounds to specific sites at the desired therapeutic rate and concentration offers a promising strategy to enhance the therapeutic efficiency of bone fracture treatment [3, 4, 5].

Biodegradable polymers used for drug encapsulation can facilitate controlled release into the tissue [6] and allow for higher drug loading capacity [7]. In particular, the development of drug‐loaded alginate (AL) bead formulations demonstrates high drug‐loading efficiency and bioavailability [8]. AL has been commonly used in bead fabrication for encapsulating drugs [9] and is widely utilized in the pharmaceutical industry due to its favorable properties such as pH‐responsive behavior [10]. The combination of gelatin (GEL) with AL can increase the performance of the drug delivery system by leveraging their individual beneficial features [11]. The addition of GEL into AL beads improves the encapsulation efficiency and prolongs the drug release [12], accelerating the maturation, mineralization, and differentiation of osteoblast cells [13, 14].

Gallic acid (GA), a major type of phenolic acid, can accelerate osteogenic differentiation of mesenchymal stem cells [15, 16]. Many studies have confirmed the potential of GA in promoting bone regeneration by inducing osteogenic differentiation. In the presence of 6.25 × 10^−^2 μg/mL sulfonamide‐based GA, osteogenesis was triggered and osteoclast differentiation was inhibited by 10 μM GA [15, 17]. Additionally, the addition of 22 μg/mL GA onto plain chitosan improves the differentiation of bone‐marrow‐derived mesenchymal stem cells through the activation of the canonical Wnt/β‐catenin signaling pathway [18].

Utilizing the advantages of AL/GEL as the encapsulating component and GA as the drug, this study aims to produce injectable beads by incorporating GA into AL/GEL microparticles and evaluate their cytocompatibility on MC3‐T3 pre‐osteoblast cells in vitro. To this end, GA‐loaded AL/GEL beads are fabricated using a simple and potentially clean extrusion dripping technique, using calcium chloride as the crosslinking agent. The study examines the loading efficiency of GA into beads, degradation, as well as the morphology, stability, chemical composition, and swelling behavior of the resulting beads. Additionally, the impact of GA‐loaded AL/GEL beads, prepared under various conditions, on cell density and viability of osteoblast cells is evaluated using MC3T3‐E1 cells.

## 2 Methodology

### 2.1 Preparation and Optimization GA‐Loaded AL/GEL Beads

Alginate (AL, Sigma‐Aldrich, medium viscosity (≥ 2000 cP, 2% (25°C)), A2033, USA) solution with 2 wt./v % concentration was prepared using deionized water solution at room temperature. An equal volume of gelatin (GEL, bovine skin, type B, Sigma‐Aldrich, G9382, Gel strength ~225 G, USA) solution with concentrations of 0.2, 0.4, 0.8, 1.2, and 2 wt./v % was added into the 2 wt./v % AL solution under continuous stirring, maintaining a 1:1 volume ratio. Calcium chloride (CaCl2) (Sigma‐Aldrich, C1016, USA) 150 mM was used as a crosslinking solution. Mixed solutions were loaded into a 10 mL syringe, and the flow rate was set to 30 mL/h. The solution was added dropwise into the calcium chloride solution with a concentration of 2 wt./v % to promote gelation. The gel beads formed were kept in the solution for an additional 10 min to be fully cured at RT. It was verified whether uniform beads were obtained, and the diameters were determined by measuring those of 50–60 collected beads using Image‐J software.

In order to prepare GA loaded beads, GA (Sigma‐Aldrich, G7384, USA) solution with 0.1, 0.5, and 1 wt.% concentration was added to the AL/GEL solution using AL and GEL solutions with the concentration of 1 and 0.2 wt/v %, respectively. The pH of the mixture was adjusted to 3.42 using acetic acid. Then, the mixed solutions were loaded into a 10 mL syringe and added dropwise into calcium chloride solution at a stirring speed of 1000 rpm for gelation. The gel beads were kept in solution for an additional 10 min. The samples prepared are introduced in Table 1.

**TABLE 1:** Composition of the prepared GA‐loaded AL/GEL beads.

Abbreviation	Alginate (AL) (wt./v%)	Gelatin (GEL) (wt./v%)	GA (GA) (wt./v%)
Unloaded AL/GEL bead	1	0.2	0
0.1% GA‐loaded AL/GEL bead	1	0.2	0.1
0.5% GA‐loaded AL/GEL bead	1	0.2	0.5
1% GA‐loaded AL/GEL bead	1	0.2	1

### 2.2 Characterization of GA Particles

Morphological analysis of GA was performed using a bright field microscope (Zeiss Axio Vert.A1 inverted microscope, Oberkochen, Germany). Particle size values of GA were measured using Image J software and graphed using GraphPad Prism.

### 2.3 Characterization of GA‐Loaded Bead

GA‐loaded beads were characterized in terms of their surface, morphology, chemical composition, and biological properties, as described below.

#### 2.3.1 Surface Characterization

Functional groups of GA‐loaded beads were characterized by using a Fourier transform infrared spectroscopy (FTIR) (Thermo Scientific Nicolet 380, FTIR Spectrometer) ranging from 4000 to 400 cm^−1^. The FTIR spectra were analyzed by using OMNIC package. Functional groups, chemical interactions, and crosslinks of GA‐loaded beads were compared with those of pure GA, AL, and GEL.

#### 2.3.2 Morphological and Chemical Composition Characterization

Morphology of selected GA loaded AL/GEL beads samples were analyzed using a scanning electron microscope (SEM) (Thermo Fisher Scientific QuattroS) operating at 10 kV. For this, beads were dried at 37°C for 1 day and mounted on an aluminum stub, through which backscattered electron (BSE) and secondary electron (SE) microscopy were performed. Elemental composition analysis was performed on various regions of beads was performed by energy‐dispersive X‐ray spectroscopy (EDX). It was verified whether uniform beads were obtained, and the diameters were determined by measuring those of 50 collected beads by using Image‐J software. The diameter of each bead was determined by manually outlining the bead boundary and applying the “Measure” function in ImageJ. The diameter values were graphed using GraphPad Prism.

#### 2.3.3 In Vitro Degradation Studies

The fabricated beads were transferred into MEM Alpha (Biowest) supplemented with 10% fetal bovine serum (FBS, Biowest) and 1% penicillin–streptomycin (PSA, Biowest), 4 mM L‐glutamine (Biowest). Varying concentrations of GA loaded into AL/GEL beads were incubated in vitro conditions at 37°C and humidified at 5% CO_2_ atmosphere to test their stability for 5 days. Morphological evaluation of GA‐loaded beads was further visualized using a bright field microscope (Zeiss Axio Vert.A1 inverted microscope, Oberkochen, Germany). A total of 5 beads were selected for each study group, and the diameter of beads was recorded on day 0, 1, 3, and 5 using the Zeiss Program (Carl Zeiss Microscope, Oberkochen, Germany) [19]. The diameter values measured at various times were graphed using GraphPad Prism.

#### 2.3.4 GA Loading Into the Beads

GA loading into beads was investigated by measuring the concentration of GA in the calcium chloride solution after the crosslinking process. This evaluation was performed by measuring the absorbance at the wavelength of 265 nm using a NanoDrop 2000/2000c UV–Vis Spectrophotometer (Thermo Scientific). The encapsulation efficiency of GA was then calculated using Equation (1) and graphed using GraphPad Prism.

(1) $$\mathrm{EE}\;\left(\%\right)=\frac{M_{0}-M_{s}}{M_{0}}\times 100$$

where EE (%) is encapsulation efficiency percentage, M_0_ is the total weight of GA in the initial slurry, and M_s_ refers to the weight of the supernatant. As such, M_0_‐M_s_ is the amount of GA in the beads. The remaining GA content in a single AL/GEL bead was calculated based on the encapsulation efficiency obtained after the production of AL/GEL beads from the GA/AL/GEL solution, as shown in Table S2. The remaining GA amount within single AL/GEL beads was calculated considering encapsulation efficiency after the production of the AL/GEL beads from GA/AL/GEL solution. GA per bead was determined as follows:

$$\mathrm{GA}\;\mathrm{per}\;\text{bead}=\left(\frac{\mathrm{EE}}{100}\times M_{0}\right)/N$$

To measure the GA release from the beads, dried GA‐loaded beads were weighed and soaked in 10 mL PBS solution (pH: 7.4). The samples were subjected to continuous agitation using a magnetic stirrer at 250 rpm. PBS samples were collected at defined time intervals, and the GA concentration in collected samples was measured by collecting the UV–Vis spectra of the solution at 265 nm by NanoDrop 2000/2000c. The cumulative amount (*GA*_*t*_) of GA in PBS was compared with the initial amount (GA0) of GA in GA‐loaded beads. The released GA from the beads was determined as follows:

(2) $$\mathrm{GA}\;\text{release}\;\left(\%\right)=\frac{{\mathrm{GA}}_{t}}{{\mathrm{GA}}_{0}}\times 100\%$$

Cumulative % GA release was graphed using GraphPad Prism.

#### 2.3.5 SEM–EDX Analysis After In Vitro Incubation of GA‐Loaded Beads With MEM Alpha Solution

After washing the beads with PBS, the fabricated beads were transferred into MEM Alpha (Biowest) supplemented with 10% fetal bovine serum (FBS, Biowest), 1% penicillin–streptomycin (PSA, Biowest), and 4 mM L‐glutamine (Biowest). GA loaded AL/GEL beads with concentrations of 10 beads/mL were prepared and then incubated under in vitro conditions at 37°C and humidified at 5% CO_2_ atmosphere to assess their stability over a 24‐h period [20]. Following day 1, the beads were collected for morphological analysis. Backscattered electron (BSE) imaging was performed on the dried GA‐loaded AL/GEL beads, and elemental composition analysis was conducted using energy‐dispersive X‐ray spectroscopy (EDX).

pH values of the samples incubated in alpha‐MEM solution were measured by a pH meter (Mettler Toledo) on days 1, 3, and 5. The pH values were demonstrated by using GraphPad Prism.

### 2.4 Cell Culture Studies

MC3T3‐E1 murine pre‐osteoblast cell line (Calvaria newborn mouse derived, ATCC CRL2593) was cultured in alpha‐MEM (Biowest) supplemented with 10% fetal bovine serum (FBS, Biowest) and 1% penicillin–streptomycin (PSA, Biowest) at 37°C under a humidified 5% CO_2_ atmosphere. Media were exchanged with fresh media every 2 days during incubation.

#### 2.4.1 Cell Density of MC3T3‐E1 Osteoblast Cell Line on the Beads

MC3T3‐E1 pre‐osteoblast cell lines were used to investigate the biocompatibility of the beads. GA‐loaded AL/GEL beads were sterilized by UV treatment for 45 min, and then fabricated AL/GEL beads loaded with varying concentrations of GA (ranging from 0 to 1 wt.%) were transferred into a cell‐seeded (3.5 × 10^5^ cells/cm^2^) well plate and incubated on top of the cells. Media were exchanged with fresh medium every 2 days. Cells were monitored during the incubation period. GA‐loaded AL/GEL bead‐treated cells were visualized with a fluorescence microscope (Zeiss Axio Vert.A1 inverted microscope for advanced routine) on days 1, 3, and 5. Three pictures from each study group were selected to measure MC3T3‐E1 cell density. The cell density of the tissue culture plate (TCP) on day 1 was considered with 100% viability (reference value).

#### 2.4.2 % Cell Viability of MC3T3‐E1pre‐Osteoblasts on the Beads

Alamar Blue assay (Invitrogen, DAL1025) was used to investigate the % cell viability of MC3T3‐E1 that was treated with GA‐loaded AL/GEL beads. In this assay, the conversion of resazurin to fluorescent resorufin by living cells indicates the metabolic activity of the cells. Firstly, the medium was replaced with fresh medium including Alamar blue solution (10%). Cells were incubated for 4 h to obtain a reduced form of the dye at 37°C and a humidified 5% CO_2_ atmosphere condition. Measurement of the reduction of Alamar blue was performed in triplicate wells. Absorbance of Alamar Blue was measured at 570 nm, with 600 nm used as a reference wavelength. The reduction of Alamar blue % of the control well (TCP) was considered as 100% (reference value). % cell viability was demonstrated using GraphPad Prism.

### 2.5 Statistical Analysis

The statistical analysis of the collected data in the present study were performed using GraphPad Prism 7 software (Boston, MA, USA). The results were analyzed by running a one‐way ANOVA followed by Dunnett's multiple comparisons test between the pairs. The average data were extracted using 3 independent experiments with *p*‐value < 0.05. The level of significance on graphs was shown at *: *p* < 0.05, **: *p* < 0.01, ***: *p* < 0.001, and ****: *p* < 0.0001.

Cohen's *d* effect sizes were calculated to evaluate the magnitude of differences between each GA group and the control (TCP). Cohen's *d* was determined using the following formula:

$$d=\frac{M_{1}-M_{2}}{{\mathrm{SD}}_{\text{pooled}}}$$

where M_1_ and M_2_ are the means of the two groups, and SDpooled is the pooled standard deviation, calculated as:

$${\mathrm{SD}}_{\text{pooled}}=\sqrt{\frac{{\mathrm{SD}}_{1}^{2}-{\mathrm{SD}}_{2}^{2}}{2}}$$

These calculations were based on the same datasets used for ANOVA and are presented in Table S1.

## 3 Results and Discussion

In this study, GA‐loaded AL/GEL beads were fabricated to investigate their physical, chemical, and biological properties. The encapsulation performance of the delivery system was evaluated, and cell culture studies were conducted to assess the effect of GA on CaP formation and cytocompatibility.

### 3.1 Optical Morphology and Surface Characterizations

**FIGURE 1:**
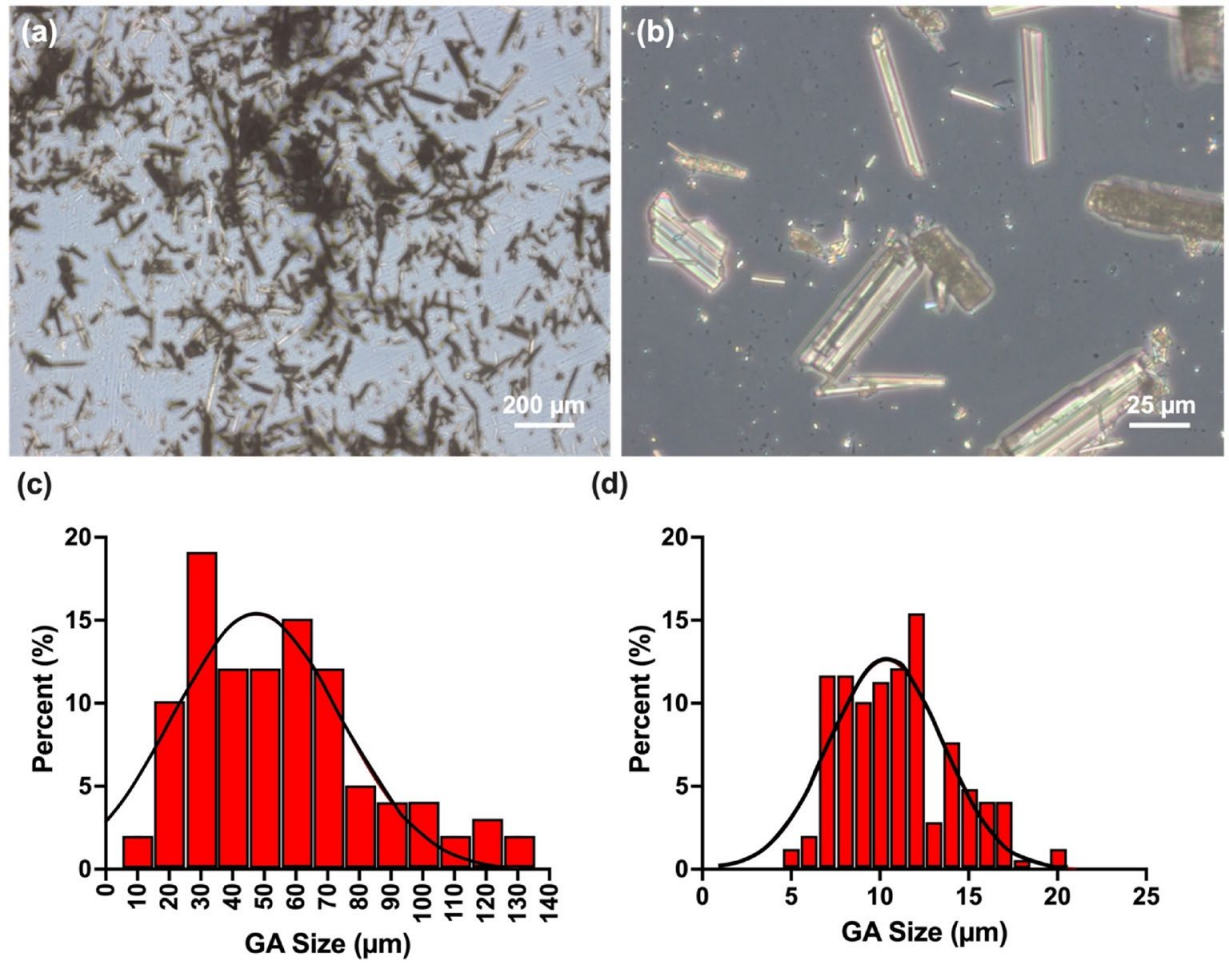
Bright field optical micrographs of GA with (a) lower magnification and (b) higher magnification, (c) Particle length distribution of GA. (d) Particle width distribution of GA.

Typical bright field optical micrographs of GA at different magnifications are shown in Figure 1a,b, and the particle length and width distribution were graphed in Figure 1c,d.

**FIGURE 2:**
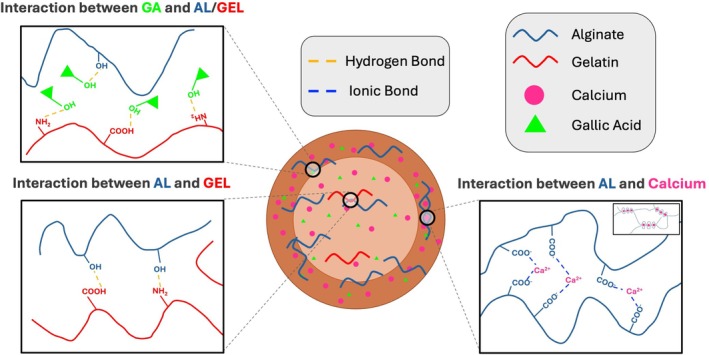
Physical and chemical interaction of AL/GEL hydrogel.

From the micrographs of Figure 1a,b, the formation of a needle‐shaped crystals of GA is evident [21]. The average length of the GA particles can be calculated to be ~45 μm. Frequency length and width distribution of GA particle size are between 10 and 129 μm and between 5 and 20 μm as shown in Figure 1c,d, respectively. Additionally, the highest length and width frequency of GA particles were detected between 30 and 60 μm and between 7 and 12 μm, respectively. The physical interaction of AL/GEL hydrogel is represented in Figure 2.

Figure 2 suggests the interaction between GA, AL, and GEL. Accordingly, GA interacts with AL and GEL via hydrogen bonding shown in green in the schematic diagram. The figure also shows the gel formation of AL with calcium ions leading to a crosslinked network. Additionally, this figure demonstrates that GEL and AL interact with each other via both hydrogen bonding and physical entanglement.

**FIGURE 3:**
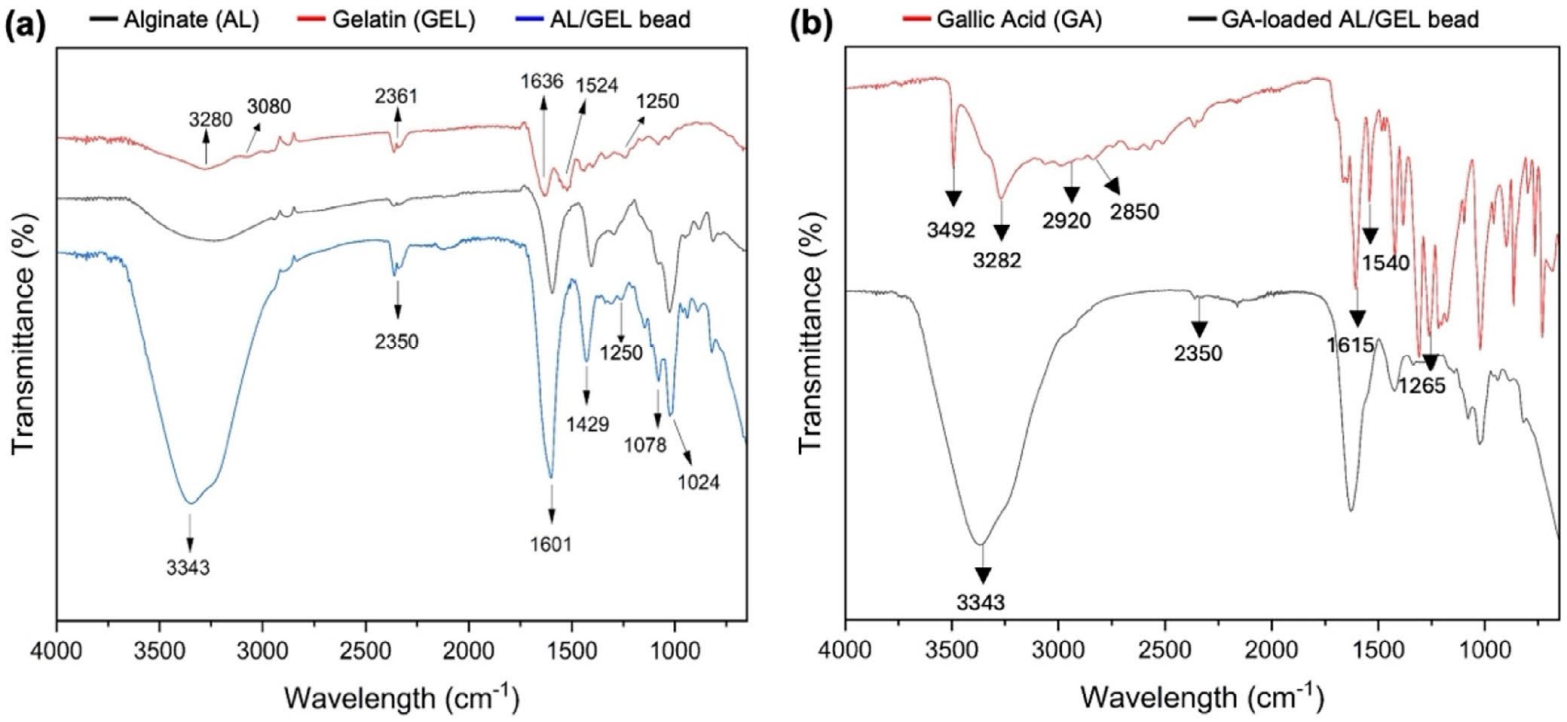
(a) FTIR spectra of AL, GEL, and AL/GEL bead. (b) FTIR spectra of GA and GA‐loaded AL/GEL bead.

Figure 3a shows the FTIR spectra of Al, GEL, and AL/GEL beads, and Figure 3b indicates those of GA and GA‐loaded AL/GEL beads.

FTIR was used to evaluate the interaction between AL and GEL after blending and subsequently crosslinking with CaCl_2_ hardening solution. The FTIR spectrum of the GEL sample shown in Figure 3a indicates the characteristic polypeptide bands of GEL, which are amide A, amide B, amide I, amide II, and amide III bands at 3280, 3080, 1636, 1524, and 1250 cm^−1^, respectively [22]. The bands at 1601 and 1429 cm^−1^ observed in the spectra of the AL/GEL beads (Figure 3a) represent the asymmetric and symmetrical vibration of the carboxyl group of AL, respectively [23, 24]. Also, the bands at 1078 and 1024 cm^−1^ of AL and AL/GEL beads observed in Figure 3a can be assigned to the stretching vibration of C‐O bonds originating from the pyranosyl ring of AL [25]. On the other hand, the peak at 1250 cm^−1^ observed in the spectrum of AL/GELan be attributed to the N‐H deformation of GEL, confirming the presence of GEL in the composite [24]. Overall, the peaks of AL/GEL beads demonstrate the presence of AL, as it is a major component of the beads with a 1.0% (w/v) concentration [26].

The FTIR spectrum of GA, shown in Figure 3b, indicates seven major peaks at the range of 3492, 3282, 2920, 2850, 1615, 1540, and 1265 cm^−1^. In the GA spectrum, peaks at 3492 and 3282 cm^−1^ of the GA spectrum are attributed to OH bonds of GA, and the bands at 2920 and 2850 cm^−1^ to stretching vibration of the aromatic CH bond [27]. Additionally, peaks at 1615 and 1540 cm^−1^ in the FTIR spectrum of GA can be assigned to stretching vibration of C=C in the aromatic ring of GA [28]. Lastly, the characteristic band of GA at 1265 cm^−1^ represents C=O stretching. Li et al. [26]. confirmed that several small bands from 3000 to 3500 cm^−1^ of GA‐loaded AL/GEL bead were sharper than they were in that of pure GA. It can be seen from the FTIR spectrum of GA‐loaded AL/GEL bead in Figure 3b that the FTIR spectrum of the AL/GEL bead did not change after loading GA. GA release during gelation of GA‐loaded AL/GEL bead might reduce the peak intensity between 3000 cm^−1^ and 3500 cm^−1^ of GA‐loaded AL/GEL bead.

### 3.2 Morphology and Particle Size of the GA/Al/Gel Beads

**FIGURE 4:**
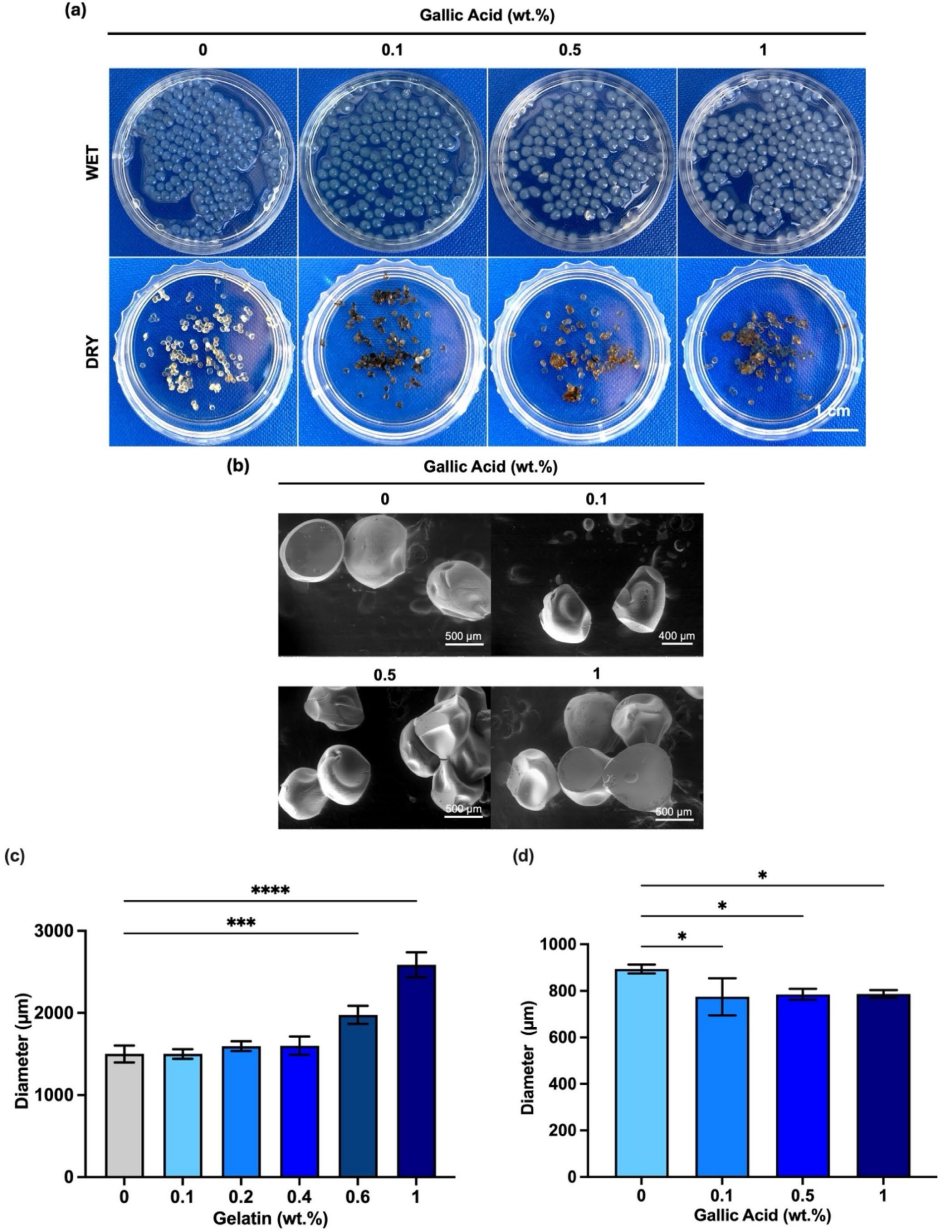
(a) Optical photos recorded on wet and dried GA‐loaded AL/GEL beads. (b) SEM images (SE mode) of GA loaded AL/GEL beads. (c) Influence of the GEL concentration on the diameter of wet beads. (***:*p* < 0.001, and ****: *p* < 0.0001 in comparison with 0% GEL group) and (d) Influence of GA quantity on the diameter of dried beads between each study group. (*: *p* < 0.05 in comparison with 0% GA group) (*n* = 50).

The most widespread way of producing AL beads is the dripping technique, where AL solution is extruded through a needle into a hardening bath to prepare ionically crosslinked AL beads [29]. In this technique, various factors such as polymer concentration and hardening solution influence the bead diameter [30]. The incorporation of GEL and GA into the AL solution affects the gel formation network and particle size of the AL bead [31]. After blending AL with GEL and GA, the size of AL/GEL beads was measured using a bright field microscope and compared among the study groups. Bead optical morphologies are exhibited in Figure 4a,b shows SEM micrographs of AL/GEL hydrogel. The effect of blending AL with GEL and GA on bead diameter is highlighted in Figure 4c,d, respectively.

According to Figure 4a, uniform spherical beads could be fabricated after the addition of GEL and GA, where wet and dry beads appear in different colors and sizes. There are no significant differences observed in terms of bead morphology due to the addition of 0.2 wt.v% GEL or GA. Dry beads were found smaller due to the release of water molecules during the dehydration process. Moreover, the dark brown bead color observed in dry beads is due to the formation of GA‐calcium chloride or GA‐AL complexes [32].

According to Figure 4b, AL/GEL beads after drying in an oven overnight at 37°C are relatively spherical with some irregularities. With the addition of GA, AL/GEL beads' degree of irregularity seemed to slightly increase. The wrinkled surface of the beads becomes more obvious as the GA content of the beads increases due to the release of more water from GA‐loaded AL/GEL beads [33].

Figure 4c shows that the increase of the GEL concentration above 0.4 wt./v% in the polymeric blend increases the size of beads. According to these observations, the GEL concentration provides a statistically significant effect on the size of beads at 0.6% and 1% GEL. Additionally, the difference in bead diameter of AL and AL/GEL is due to the inhibition of coordination between carboxyl groups of the AL chain and Ca^2+^ ions [34].

According to Figure 4d, the diameter of GA loaded AL/GEL beads could be analyzed after drying in an oven overnight at 37°C. The evaporation of water molecules resulted in a greater reduction in the diameter of GA‐loaded AL/GEL beads compared to AL/GEL beads without GA. Moreover, it is straightforward to assume that the GA addition into AL/GEL beads could enhance the hydrogen bonding between water molecules and GA within the bead [35]. Bhatia et al. [36] confirmed that GA enhanced the crosslinking density of Gel‐casein films at various loading levels, resulting in a smoother morphology. Hydrogen bonding between GA and the polymeric chains led to a more compact and denser structure. Furthermore, Yann et al. [37] showed that hydrogen bonding forms between GA and GEL polymer chains. In a similar manner, these findings show that GA‐loaded AL/GEL beads form smaller, compact, and denser microbeads compared to AL/GEL beads.

### 3.3 SEM/EDX Analysis for Compositional Analysis of GA/AL/Gel Beads

**FIGURE 5:**
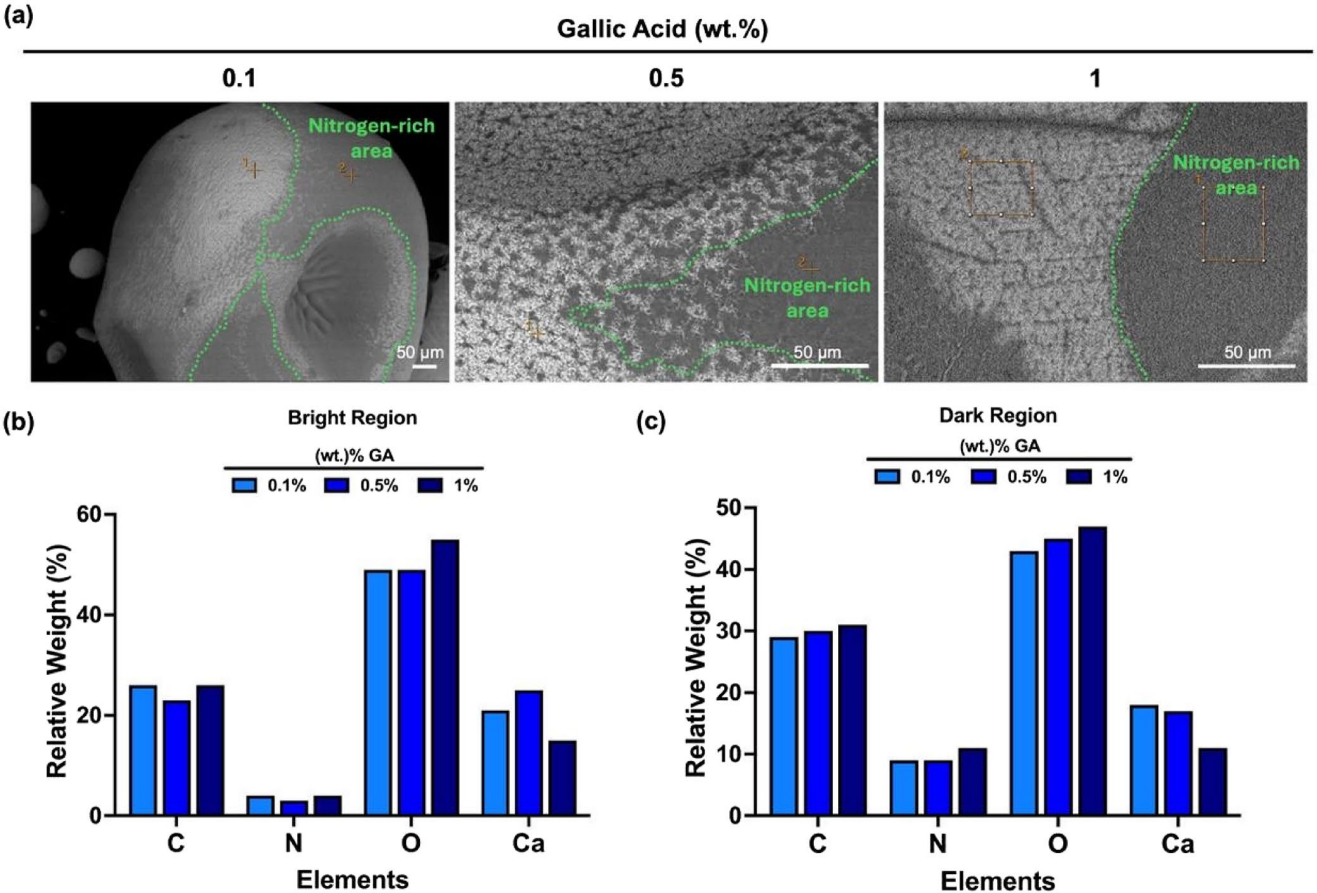
Morphology and composition of the beads (a) BSE micrograph of GA loaded AL/GEL beads. Bar graph represents distribution of each element in the (b) bright and (c) dark regions of beads.

BSE micrographs in Figure 5a–c show the morphology of GA‐loaded beads, in which the contrast of dark and bright regions indicates the presence of different concentrations of elements. As indicated in the figures, EDX analysis reveals that the darker regions could be assigned to the nitrogen‐rich components of thebeads.

In Figure 5a, SEM images in BSE mode show two distinct phases on GA‐loaded AL/GEL beads. Elemental mapping was performed on the bright and dark phases on the GA‐loaded beads. Figure 5b,c show the percentage of relative weight % of the main elements detected by EDX mapping of the bright surface and dark surface, respectively, as shown in Figure S2. The identified elements are carbon (C), oxygen (O), calcium (Ca) and nitrogen (N). Herein, Figure 5b has less N, which indicated that the bright region is more alginate‐rich. On the other hand, the dark region in Figure 5c is GEL‐rich due to higher N content. Moreover, Fang et al. [38] showed that the addition of GA increases the absorption peak of C. Additionally, the dark region of the GA‐loaded bead contains more C content compared to the bright region on the bead in Figure 5b. This finding confirms that the high content of C in the dark region indicated that GA binds to gelatine‐rich region in Figure 5c.

### 3.4 Swelling Studies, GA Loading Efficiency and GA Release Rate From the Beads

**FIGURE 6:**
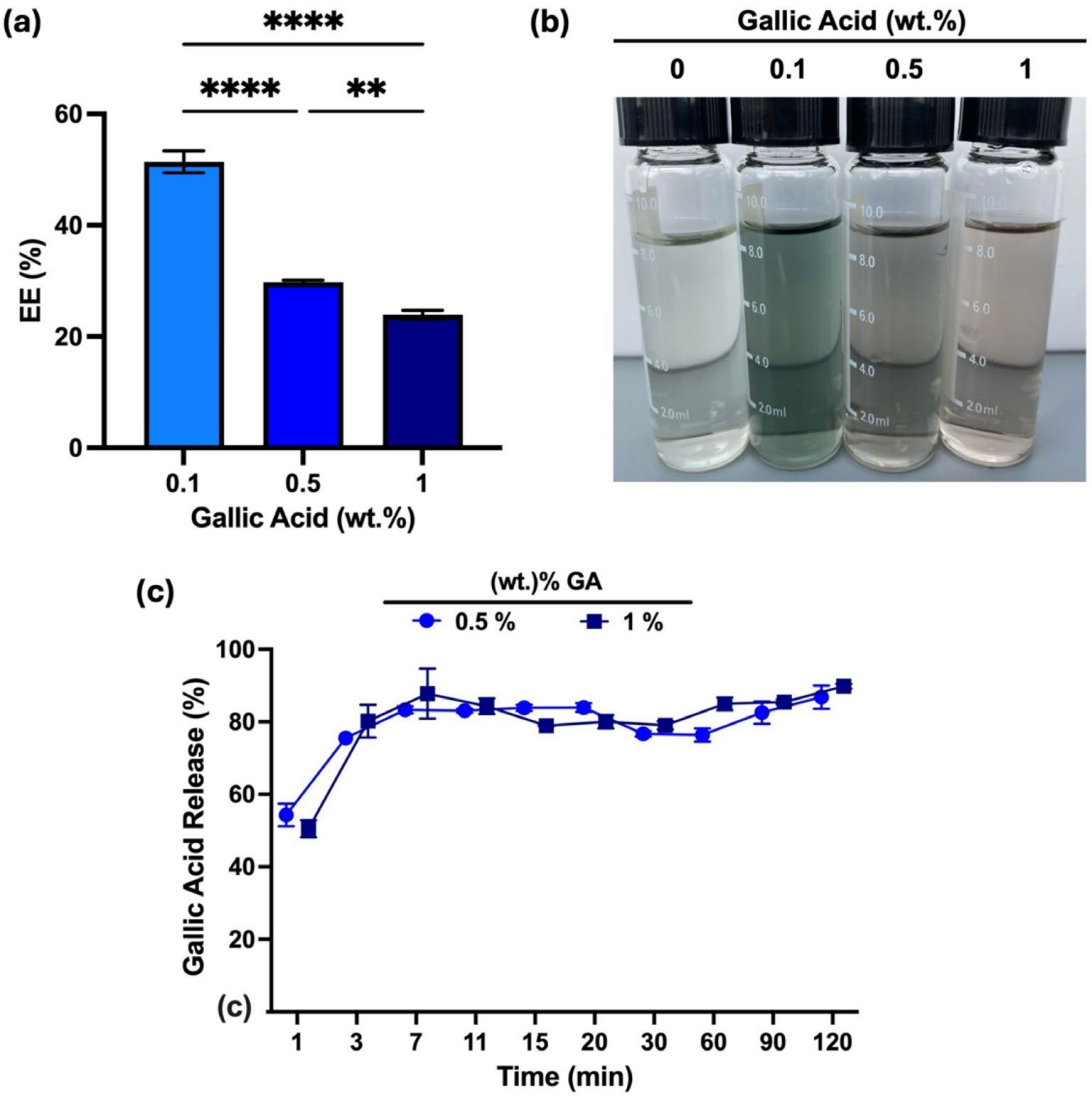
In vitro studies of beads. (a) Influence of GA (%) on encapsulation efficiency (EE) of GA‐loaded AL/GEL beads. (b) Digital photos of calcium chloride solution after crosslinking reaction of GA‐loaded AL/GEL beads. (c) GA release from AL/GEL beads in vitro (*n* = 5) (**: *p* < 0.01, and ****: *p* < 0.0001 in comparison with each study group).

Following this, the % encapsulation efficiency (EE) of GA in the beads was investigated after the addition of 0.1%, 0.5%, and 1% GA into beads in Figure 6a. The color of the hardening solution after the crosslinking process was evaluated in Figure 6b. The GA release profile from AL/GEL beads to distilled water at room temperature is represented in Figure 6c.

The diameter of beads increased from 2100 to 3000 μm after 5 days of incubation in alpha‐MEM for each study group in Figures S1 and S3. There is no correlation between GA concentration and diameter change for 5‐day incubation period of the beads. This shows that GA amount within beads does not significantly affect the swelling behavior of the beads.

Figure 6a represents that increasing the GA concentration within the beads decreases the % encapsulation efficiency. On the other hand, previous findings indicate that % encapsulation efficiency can be increased by increasing AL concentration [39]. In this study, ionic interaction between carboxylate groups from AL and divalent cations (Ca^2+^) might have decreased after the addition of GA [40]. This process is followed by the dissociation of AL and AL release into the environment, which may lead to a decrease in % encapsulation efficiency of GA in the beads.

Figure 6b shows that the color of the CaCl_2_ solution turned dark brown after the crosslinking process due to the released GA‐CaCl2 or GA‐AL complexes. GA forms a strong complex with metal ions, and the color of this complex is known as pale yellow, brown, or violet [32].

In the release profile graph, 84% GA was released from 0.5% and 1% GA‐loaded AL/GEL beads in distilled water within the first 10 min. This result is in agreement with a previous result showing 85% GA release from GA‐loaded AL beads within the first 20 min [33]. Finally, the amount of GA released from 0.1% GA‐loaded AL/GEL beads was not in the detectable concentration range of NanoDrop UV–Vis Spectrophotometer.

### 3.5 In Vitro Studies of GA‐Loaded Beads in Alpha‐MEM Solution

Simulated body fluid (SBF) is a well‐established physiological solution to stimulate in vivo biomineralization behavior of biomaterials [41]. However, the use of SBF in the biomineralization of AL beads breaks the gel network structure and leads to the rapid degradation of AL beads due to the disruption of ionic crosslinks between calcium ions and AL. Therefore, alpha MEM was used to study the mineralization of GA‐loaded AL/GEL beads [20].

**FIGURE 7:**
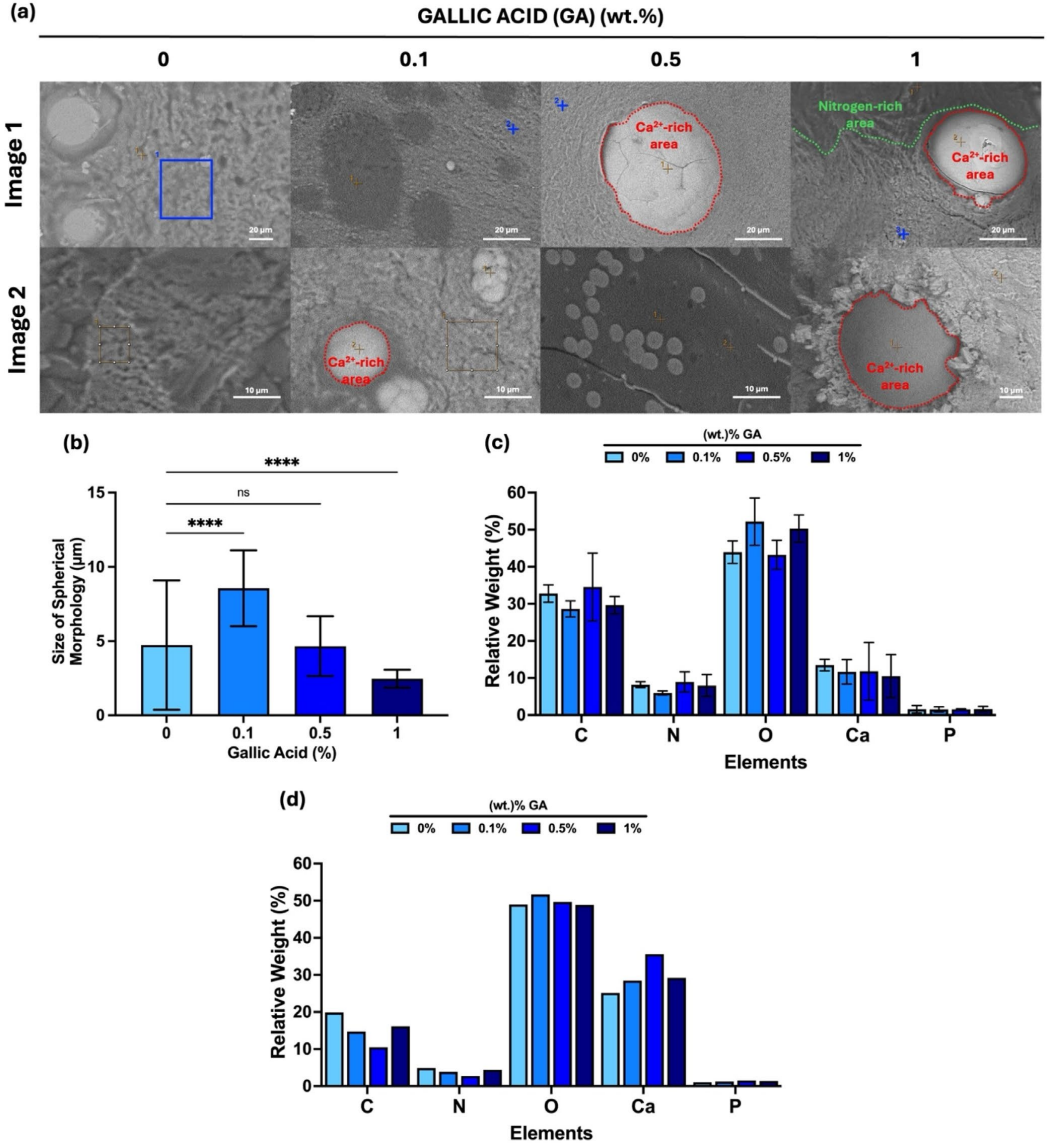
(a) SEM images (BSE mode) of GA‐loaded AL/GEL beads incubated with DMEM on day 1 (scale bar: 20 and 10 μm). (b) Size distribution of spherical morphology on 0% GA, 0.1% GA, 0.5% GA, and 1% GA loaded AL/GEL. (c) Bar graph representing a distribution of each elemental on GA loaded AL/GEL beads (blue area and dots) (*n* = 3). (d) Bar graph representing distribution of each element in the spherical morphology on GA loaded AL/GEL beads (labelled in red).

After alpha MEM incubation for 24 h, BSE imaging and EDX analysis of GA‐loaded AL/GEL beads were conducted, which is shown in Figure 7a. Particle size distribution of relatively lighter spherical microparticles found in the beads was graphed in Figure 7b. Different amounts of main elements in GA‐loaded AL/GEL beads are represented in Figure 7c. In Figure 7d, different amounts of main elements were graphed by elemental analysis of the spherical morphology of microparticles.

Xu et al. [20] observed the mineralized AL microbeads after incubating samples in DMEM solution for 7 days to stimulate in vivo biomineralization. Similarly, in Figure 7a, a spherical morphology with smooth surfaces was shown after incubating the samples in alpha MEM solution. Similar to Figure 5a,b, bright and dark surfaces on GA‐loaded beads were observed and labeled as a nitrogen‐rich and calcium‐rich areas in SEM images in Figure 7a. Additionally, mineral crystals with a spherical morphology with a size of 1–60 μm size range were observed and labeled as calcium‐rich areas on the surfaces of GA‐loaded AL/GEL beads in Figure 7a. The size of the beads was graphed as shown in 7(b).

In Figure 7c,d, elemental mapping was performed on the bead surface and the spherical morphology of microparticles. EDX mapping of the blue area and dots indicates that calcium and phosphate were deposited near the alginate membrane after incubating the samples in alpha MEM. Jerdioui et al. [42] showed that the addition of GA into nano‐hydroxyapatite (nHA)/GA composites increases the ratio of Ca/P due to the higher concentration of calcium on the surface of the composites that arises due to the interaction between GA and calcium. Similarly, elemental analysis of GA‐loaded AL/GEL in Figures 7d and S4 showed that GA has the ability to chelate with calcium, which led to the rise of calcium ion with the rise of GA content.

### 3.6 Effect of GA‐Loaded Beads on MC3T3‐E1 Osteoblast Cells

**FIGURE 8:**
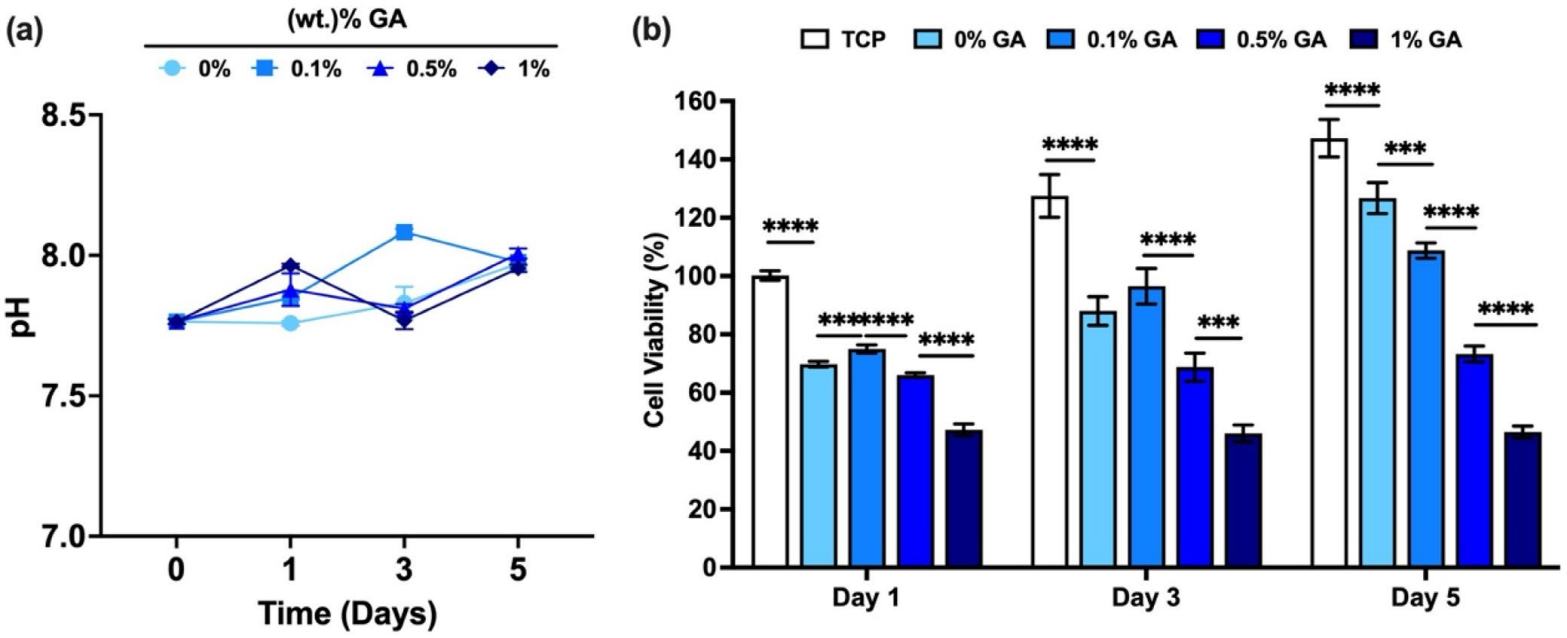
(a) pH changes in culture alpha MEM of GA‐loaded AL/GEL beads (10 beads/mL). (b) % cell viability of MC3T3‐E1 murine pre‐osteoblast cells incubated with AL/GEL beads on days 1, 3, and 5. (*n* = 5) (***:*p* < 0.001, and ****: *p* < 0.0001 in comparison with TCP: Tissue Culture Plate).

In Figure 8a, pH changes of alpha MEM were evaluated for 5 days. The effect of GA‐loaded AL/GEL beads on % cell viability was investigated with varying concentrations of GA (0 to 1 wt.%) loaded AL/GEL beads (10 beads/mL) using alamar blue cell viability assay in Figure 8b. The corresponding Cohen's d effect sizes are provided in Table S1.

Figure 8a indicates variance in pH values of alpha MEM solution from GA loaded AL/GEL beads. pH values of alpha MEM at each time point are in the range of cell culture condition and human physiological pH.

As can be seen from Figure 8b, a statistically significant effect of GA on % cell viability was observed on days 1, 3, and 5. The corresponding Cohen's *d* effect sizes in Table S1 indicate significant differences between each GA group and the control (TCP). % cell viability of MC3T3‐E1 cells was gradually decreased with an increase in GA concentration in the AL/GEL beads. % cell viability of MC3T3‐E1 osteoblast cells treated with 0.1 and 0.5 wt. % GA‐loaded AL/GEL beads was more than 70% on day 1, which indicates biocompatibility of 0, 0.1, and 0.5 wt. % GA‐loaded AL/GEL [43]. Moreover, over the incubation period of 5 days, cells proliferated on 0, 0.1, and 0.5 wt% GA‐loaded AL/GEL samples. However, 1 wt. % GA‐loaded AL/GEL beads show cytotoxicity for the MC3T3‐E1 osteoblast cell line. There are various reports showing the effect of similar beads on cell metabolic activity differently. Soran et al. [44] confirmed that GA improves the mitochondrial activity of BM‐MSC on a chitosan scaffold containing BMP‐6 (100 ng/scaffold) loaded AL beads [44]. Conversely, % cell viability of Caco‐2 cells after blank AL beads incubation did not exceed the % cell viability of Caco‐2 cells on TCP [45]. The previous study of Abnosi et al. [46] indicates that 30 μM GA reduces the % cell viability of BM‐MSC. These results suggest that AL/GEL acts as a barrier to reduce the cytotoxic effect of GA exposure on the metabolic activity of MC3T3‐E1 cells. On the other hand, GA at a concentration of 0.25 μM has been shown to increase the osteogenic ability of BM‐MSC [46]. Moreover, GA decreases the inhibition of osteogenic differentiation and reduction of % cell viability of BM‐MSC by cadmium treatment [46]. Overall, 0.1 and 0.5 wt% GA‐loaded AL/GEL beads are promising for bone regeneration applications; however, further research is required to analyze the full potential of the composites.

In Figure S3, all pictures indicate that cell density decreased as GA concentration increased. Additionally, it reveals a decrease in cell density by increasing bead concentration. Previous studies indicate that human osteosarcoma epithelial cell lines were inhibited by GA at a concentration of 0.054 mM [47]. However, the toxicity of GA was not observed on MC3T3‐E1 pre‐osteoblast cell density at a concentration of 0.1% GA‐loaded AL/GEL beads (10 beads/mL) which is an equivalent quantity corresponding to 0.39 mM GA in Table S2. Oh et al. [18] indicate that 129 μM of GA‐grafted chitosan effectively promoted osteogenic differentiation of BM‐MSC. Therefore, in the future, 0.1% GA‐loaded AL/GEL beads with a concentration of 10 beads/mL might be tested for osteogenic differentiation.

Overall, in this study, firstly, SEM–EDX indicated that GA has the ability to chelate with calcium ions. In the future, GA may be combined with resveratrol due to its bone regeneration capabilities [48] which may promote more efficient and effective bone regeneration. Furthermore, it is clear that AL and GEL require more homogeneity within the beads. To achieve this, in the future, surfactants such as tween20, tween80, or pluronic 127 may be utilized. Additionally, alternative crosslinkers should be investigated to minimize the initial burst release and to achieve a more controlled and sustained release profile of GA‐loaded AL/GEL beads. Moreover, the fabrication of smaller GA‐loaded AL/GEL beads via the electrostatic droplet generation technique may enhance % encapsulation efficiency of GA [49, 50]. Smaller GA‐loaded AL/GEL microbeads may promote improved maturation, mineralization, and differentiation of osteoblast cells. A quantitative assay like Alizarin Red eluent absorbance measurement would offer a more accurate assessment of calcium content on the bead surface. Moreover, gene expression profiling would be beneficial for analyzing gene expressions associated with bone formation, including collagen type I, calcium deposition, alkaline phosphatase, and bone sialoprotein, which could provide more conclusive evidence of the function of pre‐osteoblast cells treated with GA‐loaded AL/GEL beads. This study demonstrates that GA‐loaded AL/GEL beads can be utilized as a possible tool for supporting bone regeneration; however, further research efforts such as investigating their effect on osteogenic differentiation are required to develop their bone regeneration capabilities.

## 4 Conclusion

This study investigated morphology, chemical composition, in vitro stability, and release rate of GA from AL/GEL beads. Finally, cytocompatibility of MC3‐T3 pre‐osteoblast cells was analyzed, offering insights into their potential application in bone tissue engineering. The results demonstrate that the addition of 0.2% GEL into AL polymer solution did not affect the diameter of the resulting beads, while the release profile showed that 84% GA was released in the first 10 min. Encapsulation efficiency was found to decrease by increasing GA concentration, highlighting the challenge of maintaining high loading capacity for higher GA content. Morphological evaluation indicated that incorporating GA reduced bead diameter due to the formation of hydrogen bonds between GA and AL. However, increasing GEL concentration above 0.4 wt.% led to larger bead diameters. Moreover, GA supported localized calcium deposition, which was confirmed by elemental composition analysis. In vitro cytotoxicity studies with MC3T3‐E1 pre‐osteoblast cells demonstrated that up to 0.5 wt% GA encapsulated AL/GEL beads had high cytocompatibility. These initial results are promising for bone tissue engineering applications; however, future research is required to fully assess their potential for practical use.

## Conflicts of Interest

The authors declare no conflicts of interest.

## Supporting information


**Figure S1.** In vitro studies of beads. (a) Diameter changes in vitro condition.
**Figure S2.** Shows the cell density for all different concentrations of GA. (a) Microscopic visualization of MC3T3‐E1 pre‐osteoblast cells incubated with different concentrations of GA (0 to 1 wt.%) loaded AL/GEL beads (10 beads/mL) on days 1, 3 and 5 (scale bar: 200 μm). Cell density over time (b) 5 beads/mL, (c) 10 beads/mL, (d) 15 beads/mL. (*n* = 3) (****: *p* < 0.0001 in comparison with TCP: Tissue Culture Plate).
**Figure S3.** Digital photos of GA‐loaded AL/GEL beads in DMEM for 15 days period (scale bar: 1 cm) (*n* = 3).
**Table S1.** *p*‐values and Cohen’s d Effect Sizes for Cell Viability (%) Measurements. (Effect sizes were interpreted based on Cohen’s criteria: small (*d* ≥ 0.2), medium (*d* ≥ 0.5), and large (*d* ≥ 0.8)).
**Table S2.** GA concentrations in culture medium released from the GA‐loaded AL/GEL microbeads (10 beads/mL).

## Data Availability

Available from the corresponding author upon request.
