# Supplementary material for: Gallic Acid Loaded Alginate‐Gelatin Beads for Potential Bone Tissue Engineering Applications
Source: Biopolymers. 2025 Jun 4;116(4):e70033. doi: 10.1002/bip.70033 (PMC12136859; doi:10.1002/bip.70033)
Supplement: Supplementary file 1 — Figure S1. In vitro studies of beads. (a) Diameter changes in vitro condition. Figure S2. Shows the cell density for all different concentrations of GA. (a) Microscopic visualization of MC3T3‐E1 pre‐osteoblast cells incubated with different concentrations of GA (0 to 1 wt.%) loaded AL/GEL beads (10 beads/mL) on days 1, 3 and 5 (scale bar: 200 μm). Cell density over time (b) 5 beads/mL, (c) 10 beads/mL, (d) 15 beads/mL. (n = 3) (****: p < 0.0001 in comparison with TCP: Tissue Culture Plate). Figure S3. Digital photos of GA‐loaded AL/GEL beads in DMEM for 15 days period (scale bar: 1 cm) (n = 3). Table S1. p‐values and Cohen’s d Effect Sizes for Cell Viability (%) Measurements. (Effect sizes were interpreted based on Cohen’s criteria: small (d ≥ 0.2), medium (d ≥ 0.5), and large (d ≥ 0.8)). Table S2. GA concentrations in culture medium released from the GA‐loaded AL/GEL microbeads (10 beads/mL). [file BIP-116-e70033-s001.docx]

**Supporting Information**

The swelling behavior of the beads was examined in vitro condition in Figure S1.

**
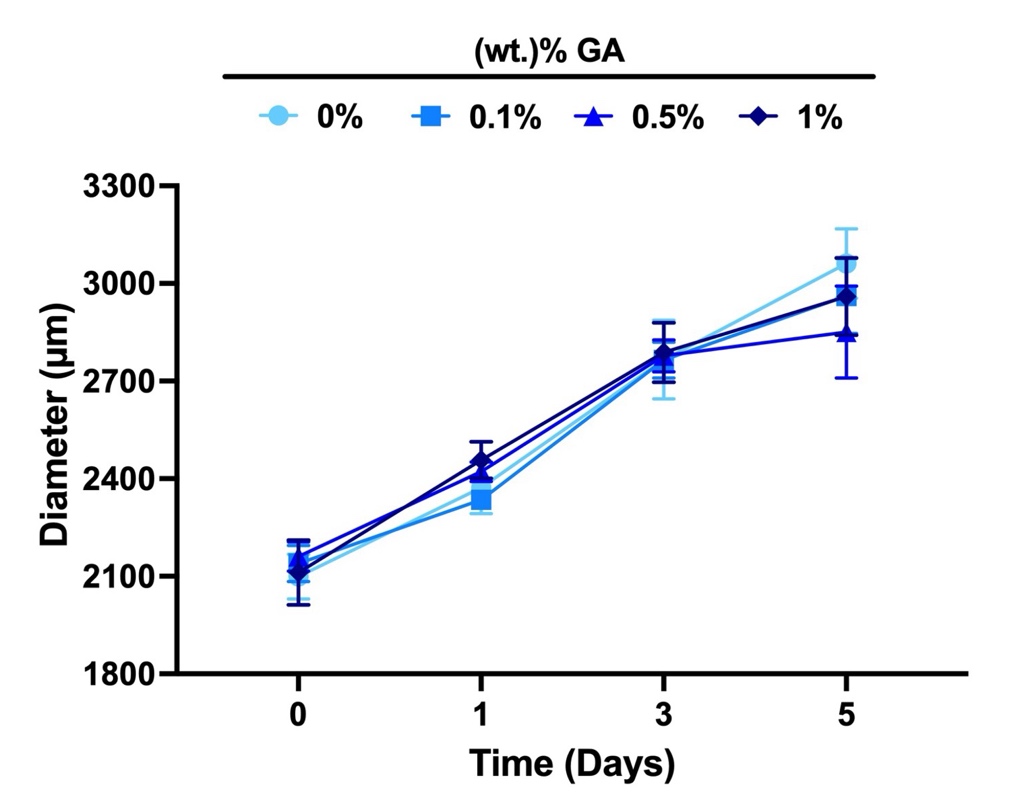
**

**Figure S1.** In vitro studies of beads. (a) Diameter changes in vitro condition.

Figure S2 shows the cell density for all different concentrations of GA.


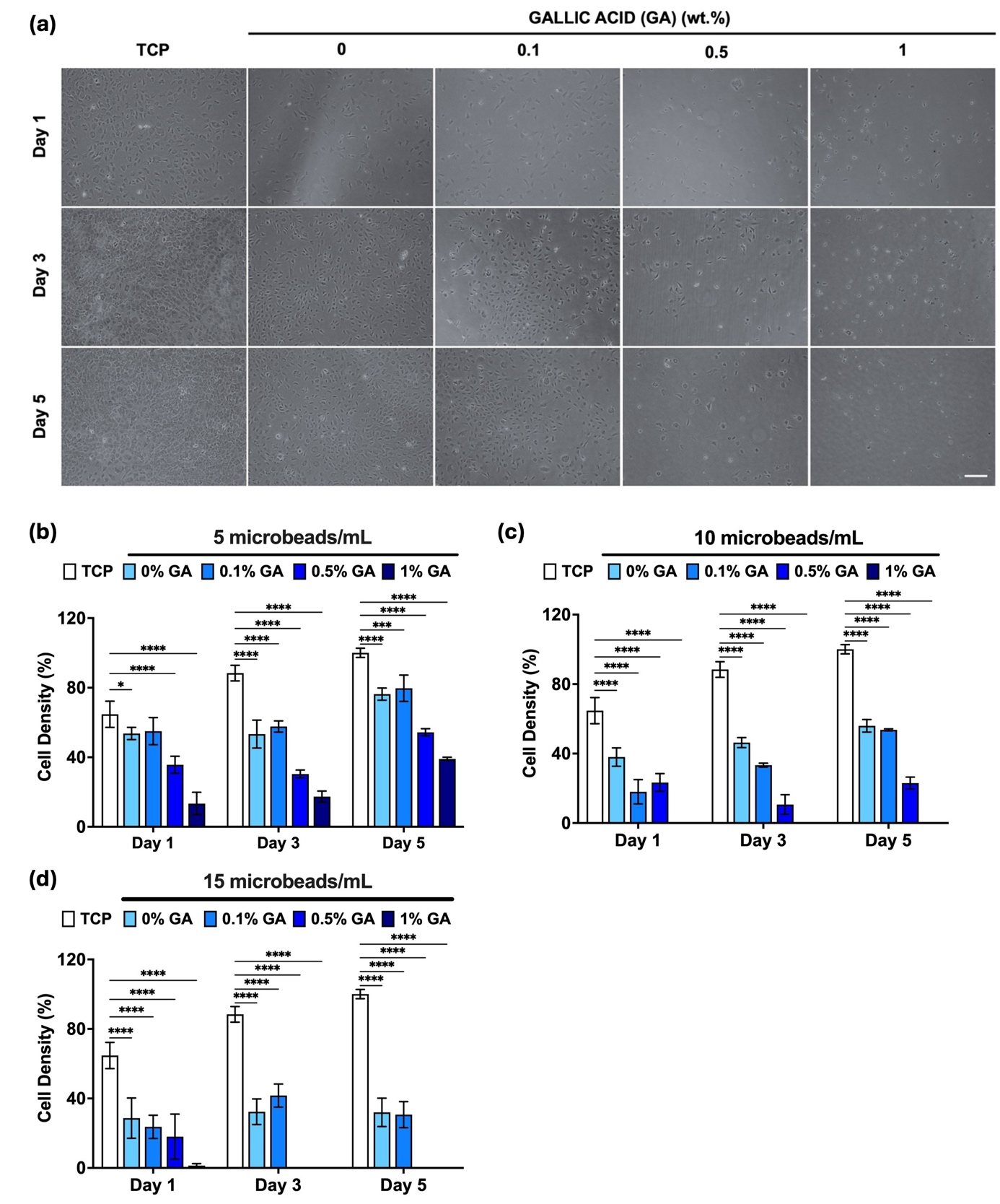


**Figure S2.** (a) Microscopic visualization of MC3T3-E1 pre-osteoblast cells incubated with different concentrations of GA (0 to 1 wt.%) loaded AL/GEL beads (10 beads/mL) on days 1, 3 and 5 (scale bar: 200 µm). Cell density over time (b) 5 beads/mL, (c) 10 beads/mL, (d) 15 beads/mL. (n=3) (****: p < 0.0001 in comparison with TCP: Tissue Culture Plate).


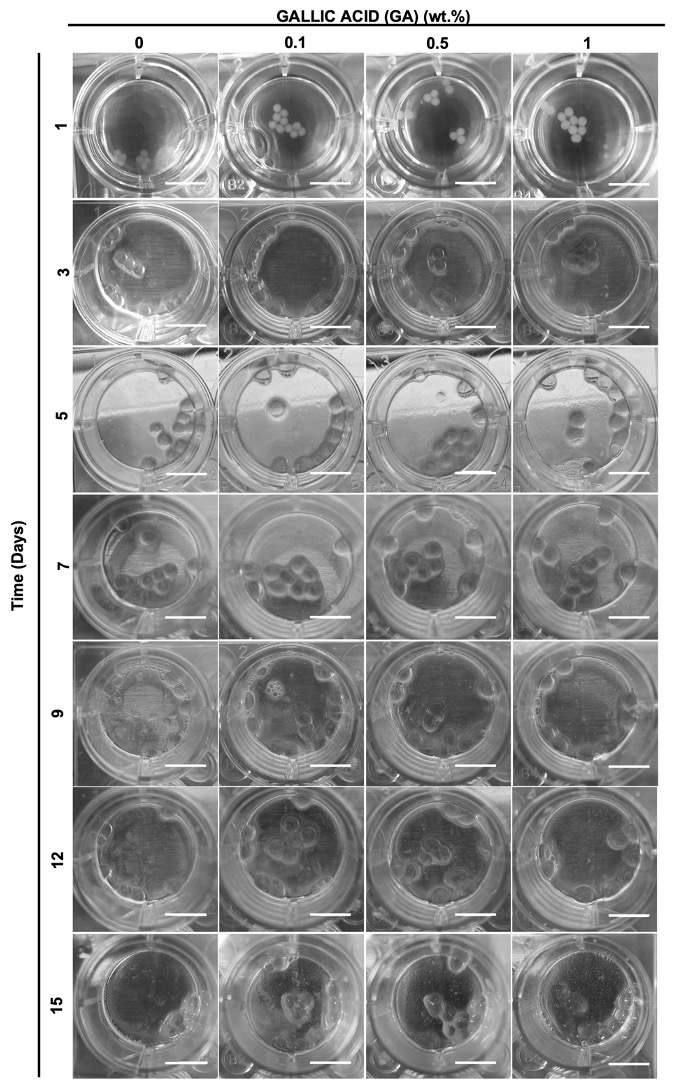


**Figure S3.** Digital photos of GA-loaded AL/GEL beads in DMEM for 15 days period (scale bar: 1 cm) (n=3).

**Table S1.** P-values and Cohen’s d Effect Sizes for Cell Viability (%) Measurements. (Effect sizes were interpreted based on Cohen’s criteria: small (d ≥ 0.2), medium (d ≥ 0.5), and large (d ≥ 0.8)).

| Day | Groups | P values | Cohen's d |
| --- | --- | --- | --- |
| 1 | TCP vs. 0% | <0,0001 | 21,89 |
|  | TCP vs. 0.1% | <0,0001 | 16,27 |
|  | TCP vs. 0.5% | <0,0001 | 25,29 |
|  | TCP vs. 1% | <0,0001 | 28,87 |
| 3 | TCP vs. 0% | <0,0001 | 6,34 |
|  | TCP vs. 0.1% | <0,0001 | 4,59 |
|  | TCP vs. 0.5% | <0,0001 | 9,45 |
|  | TCP vs. 1% | <0,0001 | 14,6 |
| 5 | TCP vs. 0% | <0,0001 | 3,49 |
|  | TCP vs. 0.1% | <0,0001 | 7,87 |
|  | TCP vs. 0.5% | <0,0001 | 15,03 |
|  | TCP vs. 1% | <0,0001 | 21,18 |

**Table S2.** GA concentrations in culture medium released from the GA-loaded AL/GEL microbeads (10 beads/mL).

| GA (µM) | | | |
| --- | --- | --- | --- |
| 0% GA-loaded microbeads (10µBeads/mL) | 0.1% GA-loaded microbeads (10µBeads/mL) | 0.5% GA-loaded microbeads (10µBeads/mL) | 1% GA-loaded microbeads (10µBeads/mL) |
| 0 µM | 0.39 mM (390 µM) | 1.24 mM (1240 µM) | 2.03 mM (2030 µM) |
